# Impact of intravenous accessibility and prehospital epinephrine use on survival outcomes of adult nontraumatic out-of-hospital cardiac arrest patients

**DOI:** 10.1186/s12873-024-00998-9

**Published:** 2024-05-06

**Authors:** Song Yi Park, Byungho Choi, Sun Hyu Kim

**Affiliations:** 1 Department of Emergency Medicine, Dong-A University College of Medicine, Dong-A University Hospital, Busan, Republic of Korea; 2 Department of Emergency Medicine, University of Ulsan College of Medicine, Ulsan University Hospital, 25, Daehakbyeongwon-ro, Ulsan, Dong-gu 44033 Republic of Korea

**Keywords:** Out-of-hospital cardiac arrest, Intravenous, Epinephrine, Cardiopulmonary resuscitation, Drug administration

## Abstract

**Background:**

This study compared out-of-hospital cardiac arrest (OHCA) patient outcomes based on intravenous (IV) access and prehospital epinephrine use.

**Methods:**

A retrospective study in Ulsan, South Korea, from January 2017 to December 2022, analyzed adult nontraumatic OHCA cases. Patients were grouped: Group 1 (no IV attempts), Group 2 (failed IV access), Group 3 (successful IV access without epinephrine), and Group 4 (successful IV access with epinephrine), with comparisons using logistic regression analysis.

**Results:**

Among 2,656 patients, Group 4 had significantly lower survival to hospital discharge (adjusted OR 0.520, 95% CI 0.346–0.782, *p* = 0.002) and favorable neurological outcomes (adjusted OR 0.292, 95% CI 0.140–0.611, *p* = 0.001) than Group 1. Groups 2 and 3 showed insignificant survival to hospital discharge (adjusted OR 0.814, 95% CI 0.566–1.171, *p* = 0.268) and (adjusted OR 1.069, 95% CI 0.810–1.412, *p* = 0.636) and favorable neurological outcomes (adjusted OR 0.585, 95% CI 0.299–1.144, *p* = 0.117) and (adjusted OR 1.075, 95% CI 0.689–1.677, *p* = 0.751). In the shockable rhythm group, Group 3 had better survival to hospital discharge (adjusted OR 1.700, 95% CI 1.044–2.770, *p* = 0.033).

**Conclusions:**

Successful IV access with epinephrine showed worse outcomes in both rhythm groups than no IV attempts. Outcomes for failed IV and successful IV access without epinephrine were inconclusive. Importantly, successful IV access without epinephrine showed favorable survival to hospital discharge in the shockable rhythm group, warranting further research into IV access for fluid resuscitation in shockable rhythm OHCA patients.

**Supplementary Information:**

The online version contains supplementary material available at 10.1186/s12873-024-00998-9.

## Background

Out-of-hospital cardiac arrest (OHCA) presents a life-threatening emergency, necessitating immediate and comprehensive intervention to enhance patient survival. Despite persistent efforts aimed at improving outcomes, the global survival rate for all attempted resuscitations of OHCA patients remains at approximately 8%, with South Korea experiencing a slightly higher rate of 9.0–9.3% [1–3].

The concept of the chain of survival includes crucial steps in the management of OHCA patients, as well as early access, early cardiopulmonary resuscitation (CPR), early defibrillation, and early advanced life support [4]. Among these steps, the prehospital administration of epinephrine is an early advanced life support intervention step performed by Emergency medical services (EMS) personnel. It is recommended for both shockable rhythms resistant to defibrillation and nonshockable rhythms [5–7], with support from numerous studies [8–10]. However, the inconsistency in determining the optimal timing for epinephrine administration within the resuscitation algorithm raises concerns, particularly regarding its potential impact on the time required to establish intravenous (IV) access [11].

The IV route assumes a critical role in swiftly and directly delivering epinephrine into the systemic circulation. Consequently, existing resuscitation guidelines advocate for attempting IV access as the initial step, considering the intraosseous (IO) route only if IV access proves unsuccessful or impractical, especially in adults with OHCA [6]. Nonetheless, securing IV access during resuscitation poses challenges, and the accessibility of the IV route may significantly influence the timely administration of epinephrine. Previous studies have demonstrated the association between the time of epinephrine administration and patient survival outcomes in OHCA [12, 13].

It is important to differentiate the survival outcomes between patient groups where EMS personnel were unable to administer epinephrine due to IV access failure and those where they chose not to use it. The former is typically examined within an intention-to-treat population. However, in certain instances, both categories of patients are lumped together as “non-epinephrine users.” Determining the appropriateness of combining these groups in an observational study is pivotal. Understanding the interplay between IV accessibility and epinephrine administration is crucial for evaluating the impact of prehospital epinephrine use on real-world emergency medical care decisions. Unfortunately, only a limited number of studies have delved into how IV accessibility influences prehospital epinephrine use and the survival outcomes of the IV-failed population of OHCA patients. Consequently, this study aimed to compare survival outcomes among adult nontraumatic OHCA patients, with a specific focus on IV accessibility and prehospital epinephrine use.

## Methods

### Study design

**Fig. 1:**
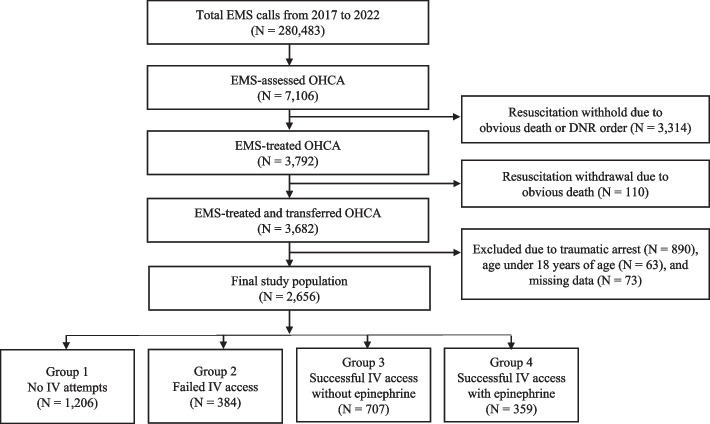
Study Population Selection and Exclusion Process. EMS, emergency medical service; OHCA, out-of-hospital cardiac arrest; DNR, do-not-resuscitate

This retrospective observational study included all adult nontraumatic OHCA patients attended to by EMS personnel in Ulsan, South Korea, from January 1, 2017, through December 31, 2022. The study aimed to evaluate and compare the survival outcomes of 4 distinct patient groups, classified based on intravenous IV accessibility and prehospital epinephrine utilization (Fig. 1). This study complied with the Declaration of Helsinki, and the protocol was approved by the Institutional Review Board of UUH with a waiver of informed consent (IRB No. UUH-IRB-2023–06-016).

- **Group 1: No IV attempts**
  EMS personnel did not attempt to establish IV access.
- **Group 2: Failed IV access**
  EMS personnel tried but could not establish IV access, so epinephrine was not administered.
- **Group 3: Successful IV access without epinephrine**
  EMS personnel established IV access but chose not to administer epinephrine.
- **Group 4: Successful IV access with epinephrine**
  EMS personnel successfully established IV access and administered epinephrine.

### Study setting

This study was conducted in Ulsan, a highly industrialized city on South Korea’s east coast, covering an area of 1,057.136 km^2^ with a population of over 1.1 million people. Ulsan’s EMS system aligns with the National EMS systems of South Korea, offering basic to intermediate-level emergency medical technician (EMT) services. These services encompass basic life support, advanced airway management, IV establishment, and fluid/drug administration. Ulsan had 30 fire stations, 32 main public ambulances with, alongside a central dispatch center in 2022 [14].

The EMS resuscitation protocol in Ulsan involves multiple dispatches, initiating on-site CPR, transporting patients to emergency departments (EDs), and maintaining continuous CPR within the ambulance. EMS personnel are mandated to sustain CPR until the return of spontaneous circulation (ROSC), the presence of death signs, or the implementation of a do-not-resuscitate (DNR) order. Declaration of death falls under the purview of hospital ED physicians. Ambulances with physician staff are not available, and advanced procedures, such as airway management, IV access, fluid and/or drug administration, and decisions regarding resuscitation withhold/withdrawal are directly overseen by medical directors, mainly emergency physicians [15]. EMS teams are typically comprised of 3 personnel (occasionally 2 personnel), with at least one EMT. They predominantly hold certifications as registered nurses or level 1 or 2 EMTs equivalent to EMT basic and intermediate levels in the United States (US). The standard protocol for OHCA cases involves dispatching multiple ambulances to ensure that at least 2 ambulances reach the scene, thus involving a minimum of 4–5 EMS personnel in resuscitation efforts. Their roles include coordination, airway management, defibrillation with chest compression, and IV access with drug administration. According to the resuscitation protocol, EMS personnel rotate every 2 min in cases of manual chest compression. Mechanical compression device usage varies by situation. In instances requiring elevator use or transfer via stairs in buildings without elevators, the EMS team immediately applies mechanical compression devices on-site. IV line access and drug administration are usually handled by registered nurses or experienced EMTs. This is performed simultaneously with prioritizing high-quality chest compressions with minimal interruption at the scene.

In November 2019, prehospital epinephrine administration for OHCA patients by EMS personnel commenced as part of a national pilot project. Previously, its use was subject to the medical director’s discretion. Even with successful IV-line establishment, fluid resuscitation alone was chosen at times, omitting epinephrine, based on the medical director’s judgment. Since 2019, certified EMS personnel can administer epinephrine under video-medical oversight after 3 days of training. Guidelines dictate that only certified EMS teams can administer IV-based epinephrine at a dosage of 1 mg every 4 min, while IO access is beyond the legal scope of practice [16]. Additionally, up to 2 IV establishment attempts are permitted, with on-scene resuscitation limited to 15 min for multiple dispatches. Epinephrine administration during ambulance transport is discouraged, emphasizing high-quality chest compressions. However, not all EMS personnel in the region are certified for prehospital epinephrine use, leading to variations in administration practices among patients [17].

### Study population

The study included all patients identified as experiencing OHCA by EMS personnel throughout the study period. The exclusion criteria were 1) resuscitation withheld or withdrawn due to obvious death signs or a DNR order, 2) presumed traumatic arrest (including intoxication and drowning), 3) patients under 18 years old.

### Data collection

The data for this study were gathered from both prehospital and hospital stages due to the absence of an integrated cardiac arrest registration system in the study region. The Ulsan Fire Agency headquarters electronically compiles prehospital data from EMS dispatches and prehospital cardiac arrest patient care reports detailing IV attempts and their outcomes. Hospital data were sourced from all 17 receiving hospital EDs in the region.

Data collection adhered to the Utstein-style reporting guidelines for cardiac arrest [18]. Confirmation of cardiac arrest relied on the absence of circulation signs. Patient variables encompassed age, sex, witnessed status, arrest location, and comorbidities (e.g., hypertension, diabetes mellitus, cerebrovascular disease, cardiovascular disease, pulmonary disease, liver disease, renal failure, and malignancy). Bystander-related variables included bystander CPR, and automated external defibrillator (AED) use. EMS-related variables covered the initial rhythm, advanced airway management, mechanical chest compression device usage, IV access attempts and success, epinephrine administration, and EMS processing times (response time, scene time, and transport time). The response time interval (RTI), scene time interval (STI), and transport time interval (TTI) were defined as the time from EMS dispatch to EMS arrival at the scene, from EMS arrival at the scene to EMS departure from the scene, and from EMS departure from the scene to EMS arrival at the ED, respectively.

Hospital variables included whether targeted temperature management (TTM) was performed, survival to hospital discharge, and favorable neurological outcomes for all patients, tracked until discharge, with neurological outcomes assessed using cerebral performance category (CPC) scales at hospital discharge. A CPC of 1 and 2 was considered a favorable outcome [19].

### Study outcomes

The primary outcome of this study was survival to hospital discharge across the 4 defined groups. The secondary outcome focused on evaluating favorable neurological outcomes.

### Statistical analysis

Descriptive statistics were employed to analyze the baseline characteristics of the study population within each group. To compare the 4 groups, either the Kruskal–Wallis test or one-way analysis of variance was utilized for continuous variables, contingent on the normality test. In cases of significant differences (*p* < 0.05), a post hoc Scheffe test was executed. The chi-squared test was applied to analyze categorical variables. Multivariable logistic regression analysis was carried out to evaluate the association between IV access, prehospital epinephrine use, and survival outcomes, calculating adjusted odds ratios (ORs) and 95% confidence intervals (CIs). Regression analysis is a statistical tool that helps in understanding how changes in one or more independent variables relate to changes in a dependent variable controlling the confounding factors. These models were adjusted for potential confounding factors, including age, sex, comorbidities, witnessed status, arrest location, bystander CPR, AED use status, initial rhythm, advanced airway management, mechanical chest compression use, and EMS processing time. Subgroup analyses were conducted to explore whether the impact of prehospital epinephrine with IV accessibility on survival outcomes varied based on the initial rhythm at the scene. Sensitivity analyses were performed to assess the robustness of our findings. We restricted the data to patients from January 1, 2020, aligning with a notable increase from 7.4% (2017–2019) to 17.8% (2020–2022) in prehospital epinephrine use due to a regional, national project. A 2-sided *p*-value of < 0.05 was considered statistically significant. All statistical analyses were carried out using SAS software (version 9.4; SAS Institute Inc., Cary, NC, USA).

## Results

During the study period, there were 280,483 EMS calls in Ulsan. Among them, 7,106 patients with OHCA were assessed by EMS personnel. A total of 2,656 patients were included in the study population and divided into 4 groups based on IV accessibility and the administration of epinephrine (Fig. 1).

### Characteristics of the study population

In the study population, attempts to establish IV access were made in 54.5% (1,450/2,656), with a success rate of 73.5% (1,066/1,450). Post-hoc analysis revealed a higher age in Group 1 compared to Group 4 (69.9 ± 15.5 years vs. 66.3 ± 15.7 years, *p* < 0.002), and higher proportion of males in Group 3 and 4 than in Group 1 and 2. The rate of arrest in public was highest in Group 3, whereas the rate of arrest in an ambulance was highest in Group 1. The RTI was longer than in Groups 3 and 4 (8.1 ± 4.3 min vs. 7.5 ± 3.4 min and 7.1 ± 3.2 min, *p* < 0.001). Group 4 exhibited a longer STI compared to Groups 3 and 2 (18.5 ± 5.1 min vs. 14.6 ± 4.7 min and 14.4 ± 5.1 min, *p* < 0.001), and Group 2 had a longer STI than Group 1 (14.4 ± 5.1 min vs. 12.4 ± 5.5 min, *p* < 0.001). Additionally, Group 3 had a longer TTI than Group 4 (6.9 ± 6.3 min vs. 5.8 ± 5.2 min, *p* < 0.010) (Table 1).

**Table 1:** Demographics and clinical characteristics of adult nontraumatic out-of-hospital cardiac arrest patients according to IV accessibility and prehospital epinephrine administration, 2017–2022

		Total	Group 1	Group 2	Group 3	Group 4	*p-*value
***Patient variables***		(*N* = 2,656)	(*N* = 1,206)	(*N* = 384)	(*N* = 707)	(*N* = 359)	
Age (years)	mean ± SD	68.9 (15.4)	69.9 (15.5)	69.1 (15.2)	68.5 (15.1)	66.3 (15.7)	0.002^*^
	median, Q1–Q3	71.0 (58.0–81.0)	73.0 (60.0–82.0)	71.0 (59.0–80.0)	70.0 (58.0–80.0)	67.0 (55.0–79.5)	
Age, distribution	≤ 39 years old	114 (4.3)	49 (4.1)	16 (4.2)	27 (3.8)	22 (6.1)	
	40 to 59 years old	603 (22.7)	251 (20.8)	82 (21.3)	173 (24.5)	97 (27.0)	
	60 to 79 years old	1,247 (46.9)	525 (43.5)	178 (46.4)	316 (44.7)	150 (41.8)	
	≥ 80 years old	692 (26.1)	381 (31.6)	108 (28.1)	191 (27.0)	90 (25.1)	
Sex (male)		1,643 (61.9)	701 (58.1)	216 (56.3)	477 (67.5)	249 (69.4)	< 0.001
Comorbidity		(*N* = 2,402)	(*N* = 1,090)	(*N* = 367)	(*N* = 598)	(*N* = 347)	
	hypertension	681 (28.4)	304 (27.9)	97 (26.4)	176 (29.4)	104 (30.0)	0.585
	diabetes mellitus	527 (21.9)	245 (22.5)	79 (21.5)	124 (20.7)	79 (22.8)	0.150
	cerebrovascular disease	169 (7.0)	74 (6.8)	27 (7.4)	42 (7.0)	26 (7.5)	0.303
	cardiovascular disease	398 (16.6)	171 (15.7)	54 (14.7)	110 (18.4)	63 (18.2)	0.261
	pulmonary disease	169 (7.0)	85 (7.8)	30 (8.2)	32 (5.4)	22 (6.3)	0.558
	liver disease	51 (2.1)	20 (1.8)	10 (2.7)	12 (2.0)	9 (2.6)	0.075
	renal failure	103 (4.3)	47 (4.3)	13 (3.5)	30 (5.0)	13 (3.7)	0.878
	malignancy	304 (12.7)	144 (13.2)	57 (15.5)	72 (12.0)	31 (8.9)	0.001
Witnessed arrest	witnessed	1,245 (46.9)	561 (46.5)	166 (43.2)	353 (49.9)	165 (46.0)	0.089
	unwitnessed	1,189 (44.8)	535 (44.4)	180 (46.9)	303 (42.9)	171 (47.6)	
	unknown	222 (8.4)	110 (9.1)	38 (9.9)	51 (7.2)	23 (6.4)	
Arrest location	public	462 (17.4)	193 (16.0)	51 (13.3)	158 (22.3)	60 (16.7)	< 0.001
	non-public	2,029 (76.4)	885 (73.4)	316 (82.3)	531 (75.1)	297 (82.7)	
	ambulance	165 (6.2)	128 (10.6)	17 (4.4)	18 (2.5)	2 (0.6)	
***Bystander variables***							
Bystander CPR	performed	1,616 (60.8)	696 (57.7)	238 (62.0)	444 (62.8)	238 (66.3)	< 0.001
	unperformed	983 (37.0)	482 (40.0)	137 (35.7)	252 (35.6)	112 (31.2)	
	unknown	57 (2.1)	28 (2.3)	9 (2.3)	11 (1.6)	9 (2.5)	
Bystander AED	applied	146 (5.5)	93 (7.7)	17 (4.4)	26 (3.7)	10 (2.8)	0.257
	not applied	2,490 (93.8)	1,098 (91.0)	367 (95.6)	679 (96.0)	346 (96.4)	
	unknown	20 (0.8)	15 (1.2)	0 (0.0)	2 (0.3)	3 (0.8)	
***EMS variables***							
Initial rhythm	shockable	445 (16.8)	166 (13.8)	58 (15.1)	157 (22.2)	64 (17.8)	0.006
	nonshockable	2,211 (83.2)	1040 (86.2)	326 (84.9)	550 (77.8)	295 (82.2)	
EMS processing time (minutes)							
RTI	mean ± SD	7.8 (3.9)	8.1 (4.3)	7.7 (3.9)	7.5 (3.4)	7.1 (3.2)	< 0.001^*^
	median, Q1–Q3	7.0 (5.0–9.0)	7.0 (5.0–9.0)	7.0 (5.0–9.0)	7.0 (5.0–9.0)	7.0 (5.0–8.0)	
STI	mean ± SD	14.1 (5.5)	12.4 (5.5)	14.4 (5.1)	14.6 (4.7)	18.3 (5.1)	< 0.001^*^
	median, Q1–Q3	14.0 (11.0–17.0)	12.0 (9.0–15.0)	14.0 (11.0–17.5)	14.0 (12.0–17.0)	17.0 (15.0–21.0)	
TTI	mean ± SD	6.6 (5.7)	6.8 (5.6)	6.2 (5.1)	6.9 (6.3)	5.8 (5.2)	0.010^*^
	median, Q1–Q3	5.0 (3.0–8.0)	5.0 (3.0–9.0)	5.0 (3.0–8.0)	5.0 (3.0–8.0)	4.0 (3.0–7.0)	
Advanced airway	no advanced airway	410 (15.4)	319 (26.5)	38 (9.9)	48 (6.8)	5 (1.4)	< 0.001
	tracheal intubation	291 (11.0)	70 (5.8)	66 (17.2)	94 (13.3)	61 (17.0)	
	supraglottic airway	1,955 (73.6)	817 (67.7)	280 (72.9)	565 (79.9)	293 (81.6)	
Mechanical CPR	applied	1,258 (47.4)	490 (40.6)	174 (45.3)	344 (48.7)	250 (69.6)	< 0.001
	not applied	1,398 (52.6)	716 (59.4)	210 (54.7)	363 (51.3)	109 (30.4)	
***Hospital variables***							
TTM	performed	32 (1.2)	9 (0.7)	6 (1.6)	11 (1.6)	6 (1.7)	0.087
	not performed	2,624 (98.8)	1,197 (99.3)	378 (98.4)	696 (98.4)	353 (98.3)	

The variables are presented as numbers (percentages). The groups were divided based on intravenous accessibility and prehospital epinephrine use. Group 1 did not have intravenous access attempted, Group 2 had a failed intravenous access attempt, Group 3 had intravenous access established but did not use epinephrine, and Group 4 had intravenous access established and epinephrine administered

*CPR* cardiopulmonary resuscitation, *AED* automated external defibrillator, *RIT* response time interval, *STI* scene time interval, *TTI* transport time interval, *TTM* targeted temperature management, *SD* standard deviation

*Four-group comparison analysis was conducted using a one-way analysis of variance (*p* < 0.05) and post-hoc analysis with the Scheffe test: Group 1 > Group 4 for age, Group 1 > Group 3 and 4 for RTI, Group 4 > Group 3 and 2 > Group 1 for STI, and Group 3 > Group 4 for TTI

### Outcomes

Table 2 presents the survival to hospital discharge and favorable neurological outcomes in the study population. Notably, Group 3 exhibited the highest ratio of survival to hospital discharge (19.8%) and favorable neurological outcomes (9.5%). Upon adjusting for potential confounders, the 4 groups displayed significant differences in survival to hospital discharge (*p* = 0.005) and favorable neurological outcomes (*p* = 0.002). However, only Group 4 demonstrated significantly lower survival to hospital discharge (adjusted OR = 0.520, 95% CI 0.346–0.782, *p* = 0.002) and favorable neurological outcomes (adjusted OR = 0.292, 95% CI 0.140–0.611, *p* = 0.001) compared to Group 1. Groups 2 and 3 showed insignificant survival to hospital discharge (adjusted OR = 0.814, 95% CI: 0.566–1.171, *p* = 0.268), (adjusted OR = 1.069, 95% CI: 0.810–1.412, *p* = 0.636, respectively) and favorable neurological outcomes (adjusted OR = 0.585, 95% CI: 0.299–1.144, p = 0.117), (adjusted OR = 1.075, 95% CI: 0.689–1.677, *p* = 0.751, respectively) compared to Group 1.

**Table 2:** Survival to discharge and favorable neurological outcomes of adult nontraumatic out-of-hospital cardiac arrest patients according to IV accessibility and prehospital epinephrine administration, 2017–2022

**Survival to discharge**					
		Unadjusted OR (95% CI)	*p*-value	Adjusted OR (95% CI)	*p*-value
Total (*N* = 2,656)	432 (16.3%)				
Group 1 (*N* = 1,206)	200 (16.6%)	Reference	0.001^*^	Reference	0.005^*^
Group 2 (*N* = 384)	53 (13.8%)	0.805 (0.581–1.117)	0.195	0.814 (0.566–1.171)	0.268
Group 3 (*N* = 707)	140 (19.8%)	1.242 (0.978–1.578)	0.076	1.069 (0.810–1.412)	0.636
Group 4 (*N* = 359)	39 (10.9%)	0.613 (0.425–0.883)	0.009	0.520 (0.346–0.782)	0.002
**Favorable neurological outcomes**					
		Unadjusted OR (95% CI)	*p*-value	adjusted OR (95% CI)	*p*-value
Total (*N* = 2,644)	162 (6.1%)				
Group 1 (*N* = 1,200)	69 (5.8%)	Reference	< 0.000^*^	Reference	0.002^*^
Group 2 (*N* = 382)	15 (3.9%)	0.670 (0.379–1.185)	0.169	0.585 (0.299–1.144)	0.117
Group 3 (*N* = 704)	67 (9.5%)	1.724 (1.215–2.446)	0.002	1.075 (0.689–1.677)	0.751
Group 4 (*N* = 358)	11 (3.1%)	0.520 (0.272–0.993)	0.048	0.292 (0.140–0.611)	0.001

The variables are presented as numbers of patients (percentages). The groups were divided based on intravenous accessibility and prehospital epinephrine use. Group 1 did not have intravenous access attempted, Group 2 had a failed intravenous access attempt, Group 3 had intravenous access established but did not use epinephrine, and Group 4 had intravenous access established and epinephrine administered. Neurological outcomes were scaled using cerebral performance categories, and categories 1 and 2 were defined as favorable neurological outcomes

*OR* odds ratio, *CI* confidence interval, *CPR* cardiopulmonary resuscitation, *AED* automated external defibrillator

^*^Four-group comparison analysis was conducted using a chi-squared test. Other *p*-values represent significance level of the 95% confidence interval. The models were adjusted for potential confounding factors, including age, sex, comorbidities, witnessed status, arrest location, bystander CPR, bystander AED use status, initial rhythm, advanced airway management, mechanical chest compression use, and EMS processing time

Table 3 presents the subgroup analysis to examine the survival outcomes of the 4 groups based on the initial rhythm at the scene. In the shockable rhythm group, a significant difference was observed among the 4 groups in terms of survival to hospital discharge (*p* < 0.001) and favorable neurological outcomes (*p* = 0.003). Group 3 demonstrated a favorable outcome in survival to hospital discharge (adjusted OR = 1.700, 95% CI: 1.044–2.770, *p* = 0.033), whereas Group 4 exhibited significantly lower survival to hospital discharge (adjusted OR = 0.391, 95% CI: 0.195–0.784, *p* = 0.008) and favorable neurological outcomes (adjusted OR = 0.294, 95% CI: 0.131–0.662, *p* = 0.003) (Table 3). In contrast, the nonshockable rhythm group displayed no significant differences among the 4 groups in terms of survival to hospital discharge (*p* = 0.052) or favorable neurological outcomes (*p* = 0.409).

**Table 3:** Survival to discharge and favorable neurological outcomes according to the initial rhythm at the scene of adult nontraumatic out-of-hospital cardiac arrest patients according to IV accessibility and prehospital epinephrine administration, 2017–2022

**Shockable rhythm at the scene**					
**Survival to discharge**					
	Survival	Unadjusted OR (95% CI)	*p-*value	Adjusted OR (95% CI)	*p-*value
Total (*N* = 445)	197 (44.3%)				
Group 1 (*N* = 166)	70 (42.2%)	Ref	< 0.001^*^	Ref	< 0.00^*^
Group 2 (*N* = 58)	22 (37.9%)	0.838 (0.454–1.548)	0.572	0.855 (0.436–1.678)	0.649
Group 3 (*N* = 157)	89 (56.7%)	1.795 (1.155–2.790)	0.009	1.700 (1.044–2.770)	0.033
Group 4 (*N* = 64)	16 (25.0%)	0.457 (0.240–0.871)	0.017	0.391 (0.195–0.784)	0.008
**Favorable neurological outcomes**					
	Favorable	Unadjusted OR (95% CI)	*p-*value	Adjusted OR (95% CI)	*p-*value
Total (*N* = 440)	134 (30.5%)				
Group 1 (*N* = 165)	52 (31.5%)	Ref	0.002^*^	Ref	0.003^*^
Group 2 (*N* = 56)	11 (19.6%)	0.531 (0.254–1.109)	0.092	0.514 (0.231–1.146)	0.104
Group 3 (*N* = 155)	61 (39.4%)	1.410 (0.890–2.234)	0.143	1.148 (0.687–1.918)	0.598
Group 4 (*N* = 64)	10 (15.6%)	0.402 (0.190–0.852)	0.017	0.294 (0.131–0.662)	0.003
**Nonshockable rhythm at the scene**					
**Survival to discharge**					
	Survival	Unadjusted OR (95% CI)	*p-*value	Adjusted OR (95% CI)	*p-*value
Total (*N* = 2,211)	235 (10.6%)				
Group 1 (*N* = 1,040)	130 (12.5%)	Ref	0.052^*^	Ref	0.258^*^
Group 2 (*N* = 326)	31 (9.5%)	0.736 (0.487–1.112)	0.145	0.800 (0.519–1.234)	0.313
Group 3 (*N* = 550)	51 (9.3%)	0.715 (0.508–1.007)	0.055	0.786 (0.547–1.129)	0.192
Group 4 (*N* = 295)	23 (7.8%)	0.592 (0.372–0.941)	0.027	0.648 (0.397–1.058)	0.083
**Favorable neurological outcomes**					
	Favorable	Unadjusted OR (95% CI)	*p-*value	Adjusted OR (95% CI)	*p-*value
Total (*N* = 2,204)	28 (1.3%)				
Group 1 (*N* = 1,035)	17 (1.6%)	Ref	0.409^*^	Ref	0.603^*^
Group 2 (*N* = 326)	4 (1.2%)	0.744 (0.249–2.227)	0.597	0.893 (0.280–2.847)	0.848
Group 3 (*N* = 549)	6 (1.1%)	0.662 (0.259–1.688)	0.387	0.842 (0.307–2.310)	0.738
Group 4 (*N* = 294)	1 (0.3%)	0.204 (0.027–1.542)	0.124	0.238 (0.030–1.893)	0.175

The variables are presented as numbers of patients (percentages). The groups were divided based on intravenous accessibility and prehospital epinephrine use. Group 1 did not have intravenous access attempted, Group 2 had a failed intravenous access attempt, Group 3 had intravenous access established but did not use epinephrine, and Group 4 had intravenous access established and epinephrine administered. Neurological outcomes were scaled using cerebral performance categories, and categories 1 and 2 were defined as favorable neurological outcomes

*OR* odds ratio, *CI* confidence interval, *CPR* cardiopulmonary resuscitation, *AED* automated external defibrillator

^*^Four-group comparison analysis was conducted using a chi-squared test. The models were adjusted for potential confounding factors, including age, sex, comorbidities, witnessed status, arrest location, bystander CPR, bystander AED use status, initial rhythm, advanced airway management, mechanical chest compression use, and EMS processing time

In the sensitivity analysis of data from 2020 through 2022. Group 4 consistently exhibited lower likelihoods of survival to hospital discharge and favorable neurological outcomes, especially in the initial shockable rhythm subgroup (see Supplementary tables).

## Discussion

This study aimed to compare survival outcomes among adult nontraumatic OHCA patients, considering variations in IV accessibility and prehospital epinephrine use. The key findings were that Group 4 (successful IV access with epinephrine) exhibited significantly lower survival to hospital discharge and favorable neurological outcomes compared to Group 1 (no IV attempts). However, the study could not establish the significance of survival to hospital discharge and favorable neurological outcomes in Group 2 (failed IV access) and Group 3 (successful IV access without epinephrine) compared to Group 1. These findings were consistent within the shockable rhythm group, with the exception of Group 3, which demonstrated improved survival to hospital discharge outcomes compared to Group 1. Conversely, in the nonshockable rhythm group, no significant differences were observed across the 4 groups concerning both survival to hospital discharge and favorable neurological outcomes.

In this study, EMS personnel achieved an IV access success rate of 73.5%, a metric that varies across studies. For instance, a US study reported a 49% success rate on the first attempt [20], whereas a UK study reported an 81.6% success rate [21]. This variability is attributed to multiple factors, such as patient-related issues (e.g., collapsed veins, obesity, and fragile skin) and environmental challenges (e.g., limited space, moving ambulances, poor lighting, and difficult patient positioning) [22, 23]. In Ulsan, EMS personnel appear to consider patient age and RTI when deciding on IV attempts, contributing to a relatively higher IV success rate. There are no specific indications for IV access candidates within the local EMS guidelines. However, the mean age of the IV-attempted groups (Groups 2, 3, and 4) was lower than that of the no IV-attempted group (Group 1), and the IV-attempted groups had a shorter RTI. Despite a high IV success rate, the epinephrine administration rate was low, at 24.8%. Our research findings suggest that if epinephrine administration is not intended, there seems to be little benefit in pursuing IV line access. Moreover, forcefully attempting IV access in situations where success rates are low appears unwarranted, as evidenced by the lack of notable differences in survival odds between Group 1 and Group 3, as well as between Group 1 and Group 2.

Group 4 exhibited significantly lower rates of survival to hospital discharge and favorable neurological outcomes compared to Group 1. One of the potential causes of these outcomes is prolonged STI. The STI in Group 4 was longer than that of the other Groups by approximately 4 min. These findings are consistent with recent research conducted in Korea, including Ulsan [24, 25]. While the local guideline recommends an STI of within 15 min, the average STI in Group 4 was 18.3 min, exceeding the recommended time frame. This delay is likely attributed to the time taken to ask for medical direction and epinephrine administration. The association between delayed epinephrine administration, longer STIs, and lower survival odds has been well-established [26–28]. For EMS-treated adult OHCA patients with an initial nonshockable rhythm, each minute delay from EMS arrival to epinephrine administration was linked to a 4% decrease in survival odds (OR = 0.96, 95% CI: 0.95–0.98) [29]. This finding emphasizes the critical need to balance successful IV access and epinephrine administration with the imperative of minimizing STIs. The consideration of IO access in local guidelines emerges as a noteworthy alternative when expedited epinephrine administration is warranted. Considering protocolizing the request for medical direction when administering epinephrine is also an option. Another factor contributing to the lower survival outcomes observed in Group 4 is the bystander AED application rate. The chain of survival relies on a series of interconnected steps, and optimal performance of the preceding steps is essential for favorable outcomes. In Group 4, the application rate of bystander AED was the lowest among the 4 groups, at 2.8%. Bystander AED usage rates vary widely, ranging from 2 to 37% [30]. However, the incidence of cardiac arrest in public locations in Group 4 was 16.7%, the second highest among the groups. This disparity between the incidence of arrest in public and the low application rate of bystander AED warrants further investigation. Future research should explore whether the low bystander AED usage rate in the Ulsan region is due to issues related to accessibility or education.

Prehospital epinephrine use holds significance in the nonshockable rhythms group, where defibrillation efficacy may be limited compared to the shockable rhythm group, emphasizing rapid defibrillation [9, 31]. However, our study revealed no significant differences in the nonshockable rhythm group concerning survival to hospital discharge and favorable neurological outcomes. This suggests that factors beyond the scope of this study, including patient-specific considerations, quality of bystander CPR, and in-hospital medical interventions, may have influenced the outcomes. Additionally, the uniformity of the treatment protocols, such as the OHCA resuscitation algorithm, may have played a role in moderating the observed outcome differences across the 4 groups, akin to a dilution effect [32]. The consistent application of this algorithm across the 4 groups might have influenced or tempered the observed outcome differences, potentially minimizing their impact on the overall study outcomes.

It is important to acknowledge several limitations in this study. Firstly, the retrospective design, although valuable for leveraging existing data, introduced inherent constraints, including reliance on historical records, potential information gaps, and issues of causality. Particularly, in the case of Group 3, it remains unclear whether the observed survival outcome resulted from the absence of epinephrine administration or prompt transfer without administering epinephrine, leading to ROSC. Secondly, unmeasured confounding variables may have influenced the outcomes, with crucial factors such as the first IV attempt time and prehospital ROSC time omitted from the analysis. Variables like initial epinephrine administration time, total epinephrine dose, and fluid resuscitation volume could also have impacted the outcomes but were not considered. While the time from collapse may have also influenced the survival of OHCA patients, its measurement was challenging and, therefore, could not be included in the analysis. The decision to forgo epinephrine administration during transport is an additional factor that could have influenced the results. In-hospital resuscitations were not factored into our analysis. Thirdly, we utilized regression analysis to examine the influence of IV line accessibility and epinephrine administration on survival outcomes while controlling for significant variables (age, sex difference, arrest location, etc.). However, we recognize that this regression model was not flawless. For instance, within the chain of survival, steps such as EMS activation, bystander AED use, and bystander CPR play pivotal roles. Despite a gradual improvement in the rate of bystander CPR performance in our study, the utilization of bystander AED remained notably low. In the regression analysis, these factors were presumed and treated independently. However, we acknowledge that we did not investigate the impact of survival outcomes when the preceding factors were not appropriately performed. It is also possible that the ambulance arrest rate in Group 1 was not fully controlled in the regression model. Finally, it is crucial to note that the findings may not be generalizable to other populations with different EMS systems. While the consistency of our findings has been supported by other studies in the country [25].

## Conclusion

Our study underscores a complex relationship between IV access attempts, epinephrine administration, and OHCA patients. Specifically, patients with successful IV access followed by epinephrine showed significantly inferior outcomes in both survival to hospital discharge and favorable neurological outcomes compared to those with no attempts at IV access. The outcomes of patients with failed IV access and successful IV access without epinephrine were inconclusive, suggesting the need for further investigation. Despite the potential financial investment required for prehospital care, our study indicates that its impact may be limited. In certain aspects, it could even be detrimental to survival outcomes. These findings could provide insights into where to focus efforts within the chain of survival for OHCA patients.

### Supplementary Information

Supplementary Material 1.

## Data Availability

The datasets presented in this article are not readily available because some of datasets used in this study belong to the Ulsan Fire Agency. Requests to access the datasets should be directed to the Ulsan Fire Agency.

## Abbreviations

OHCA: Out-of-hospital cardiac arrest
IV: Intravenous
IO: Intraosseous
EMS: Emergency medical services
EMT: Emergency medical technician
CPR: Cardiopulmonary resuscitation
ED: Emergency department
ROSC: Return of spontaneous circulation
DNR: Do-not-resuscitate
AED: Automated external defibrillator
RTI: Response time interval
STI: Scene time interval
TTI: Transport time interval
TTM: Targeted temperature management
CPC: Cerebral performance category
ORs: Odds ratios
CIs: Confidence intervals
