# Supplementary material for: Impact of intravenous accessibility and prehospital epinephrine use on survival outcomes of adult nontraumatic out-of-hospital cardiac arrest patients
Source: BMC Emerg Med. 2024 May 6;24:79. doi: 10.1186/s12873-024-00998-9 (PMC11071239; doi:10.1186/s12873-024-00998-9)
Supplement: Supplementary file 1 — Supplementary Material 1. [file 12873_2024_998_MOESM1_ESM.docx]

Supplementary Table 1. Demographics and clinical characteristics of adult nontraumatic out-of-hospital cardiac arrest patients according to IV accessibility and prehospital epinephrine administration, 2020–2022

|  |  | Total | Group 1 | Group 2 | Group 3 | Group 4 | p*-*value |
| --- | --- | --- | --- | --- | --- | --- | --- |
| ***Patient variables*** |  | (N = 1561) | (N = 691) | (N = 191) | (N = 401) | (N = 278) |  |
| Age (years) | mean ± SD | 70.1 (15.2) | 71.0 (15.2) | 70.5 (14.6) | 70.1 (14.4) | 67.3 (16.3) | 0.007^*^ |
|  | median, Q1–Q3 | 72.0 (60.0– 82.0) | 73.0 (61.0– 83.0) | 73.0 (61.0– 81.0) | 73.0 (61.0– 81.0) | 69.0 (56.0–81.0) |  |
| Age, distribution | ≤ 39 years old | 62 (4.0) | 25 (3.6) | 7 (3.7) | 11 (2.7) | 19 (6.8) |  |
|  | 40 to 59 years old | 302 (19.3) | 122 (17.7) | 35 (18.3) | 82 (20.5) | 67 (24.1) |  |
|  | 60 to 79 years old | 708 (45.4) | 314 (45.4) | 89 (46.6) | 189 (47.1) | 112 (40.3) |  |
|  | ≥ 80 years old | 489 (31.3) | 230 (33.3) | 60 (31.4) | 119 (29.7) | 80 (28.8) |  |
| Sex (male) |  | 920 (58.9) | 369 (53.4) | 95 (49.7) | 268 (66.8) | 188 (67.6) | <0.001 |
| Comorbidities | hypertension | 440 (28.4) | 198 (28.7) | 48 (22.3) | 109 (30.4) | 85 (29.4) |  |
|  | diabetes mellitus | 356 (22.9) | 160 (23.2) | 53 (24.7) | 78 (21.8) | 65 (22.5) | 0.486 |
|  | cerebrovascular disease | 99 (6.4) | 43 (6.2) | 15 (7.0) | 19 (5.3) | 22 (7.6) | 0.294 |
|  | cardiovascular disease | 243 (15.7) | 100 (14.5) | 30 (14.0) | 59 (16.5) | 54 (18.7) | 0.784 |
|  | pulmonary disease | 105 (6.8) | 52 (7.5) | 11 (5.1) | 22 (6.1) | 20 (6.9) | 0.406 |
|  | liver disease | 38 (2.4) | 14 (2.0) | 9 (4.2) | 6 (1.7) | 9 (3.1) | 0.096 |
|  | renal failure | 71 (4.6) | 30 (4.3) | 10 (4.7) | 20 (5.6) | 11 (3.8) | 0.525 |
|  | malignancy | 200 (12.9) | 93 (13.5) | 39 (18.1) | 45 (12.6) | 23 (8.0) | 0.752 |
| Witnessed arrest | witnessed | 727 (46.6) | 326 (47.2) | 78 (40.8) | 203 (50.6) | 120 (43.2) | 0.904 |
|  | unwitnessed | 748 (47.9) | 321 (46.5) | 99 (51.8) | 181 (45.1) | 147 (52.9) | 0.036 |
|  | unknown | 86 (5.5) | 44 (6.4) | 14 (7.3) | 17 (4.2) | 11 (4.0) | 0.178 |
| Arrest location | public | 237 (15.2) | 95 (13.7) | 22 (11.5) | 82 (20.4) | 38 (13.7) | <0.001 |
|  | non-public | 1227 (78.6) | 519 (75.1) | 162 (84.8) | 306 (76.3) | 240 (86.3) |  |
|  | ambulance | 97 (6.2) | 77 (11.1) | 7 (3.7) | 13 (3.2) | 0 (0.0) |  |
| ***Bystander variables*** |  |  |  |  |  |  |  |
| Bystander CPR | performed | 899 (57.6) | 362 (52.4) | 110 (57.6) | 242 (60.3) | 185 (66.5) | 0.052 |
|  | unperformed | 639 (40.9) | 323 (46.7) | 77 (40.3) | 153 (38.2) | 86 (30.9) |  |
|  | unknown | 23 (1.5) | 6 (0.9) | 4 (2.1) | 6 (1.5) | 7 (2.5) |  |
| Bystander AED | applied | 62 (4.0) | 35 (5.1) | 4 (2.1) | 17 (4.2) | 6 (2.2) | <0.001 |
|  | not applied | 1492 (95.6) | 653 (94.5) | 187 (97.9) | 382 (95.3) | 270 (97.1) |  |
|  | unknown | 7 (0.4) | 3 (0.4) | 0 (0.0) | 2 (0.5) | 2 (0.7) |  |
| ***EMS variables*** |  |  |  |  |  |  |  |
| Initial rhythm | shockable | 220 (14.1) | 83 (12.0) | 21 (11.0) | 77 (19.2) | 39 (14.0) | <0.001 |
|  | nonshockable | 1341 (85.9) | 608 (88.0) | 170 (89.0) | 324 (80.8) | 239 (86.0) |  |
| EMS processing time (minutes) | |  |  |  |  |  |  |
| RTI | mean ± SD | 8.0 (3.8) | 8.2 (4.1) | 8.2 (4.0) | 7.9 (3.6) | 7.2 (2.8) | 0.002^*^ |
|  | median, Q1–Q3 | 7.0 (6.0–9.0) | 7.0 (6.0–9.0) | 7.0 (6.0–9.0) | 7.0 (6.0–9.0) | 7.0 (5.0–8.0) |  |
| STI | mean ± SD | 14.8 (5.5) | 12.9 (5.5) | 15.5 (5.1) | 15.1 (4.8) | 18.4 (4.7) | <0.00^*^ |
|  | median, Q1–Q3 | 14.0 (11.0– 18.0) | 12.0 (10.0– 15.0) | 15.0 (12.0– 18.0) | 15.0 (12.0– 17.0) | 18.0 (16.0–20.0) |  |
| TTI | mean ± SD | 6.9 (5.9) | 7.0 (5.7) | 6.6 (5.0) | 7.5 (6.7) | 6.1 (5.5) | 0.015^*^ |
|  | median, Q1–Q3 | 5.0 (3.0–9.0) | 5.0 (3.0–9.0) | 5.0 (3.0–8.0) | 5.0 (4.0–9.0) | 4.0 (3.0–7.0) |  |
| Advanced airway | no advanced airway | 156 (10.0) | 132 (19.1) | 6 (3.1) | 16 (4.0) | 2 (0.7) | <0.001 |
|  | tracheal intubation | 80 (5.1) | 12 (1.7) | 10 (5.2) | 24 (6.0) | 34 (12.2) |  |
|  | supraglottic airway | 1325 (84.9) | 547 (79.2) | 175 (91.6) | 361 (90.0) | 242 (87.1) |  |
| Mechanical CPR | applied | 1063 (68.1) | 414 (59.9) | 141 (73.8) | 275 (68.6) | 233 (83.8) | <0.001 |
|  | not applied | 498 (31.9) | 277 (40.1) | 50 (26.2) | 126 (31.4) | 45 (16.2) |  |
| ***Hospital variables*** |  |  |  |  |  |  |  |
| TTM | performed | 16 (1.0) | 4 (0.6) | 0 (0.0) | 7 (1.7) | 5 (1.8) | 0.078 |
|  | not performed | 1529 (99.0) | 687 (99.4) | 191 (100.0) | 394 (98.3) | 273 (98.2) |  |

The variables are presented as numbers (percentages). The groups were divided based on intravenous accessibility and prehospital epinephrine use. Group 1 did not have intravenous access attempted, Group 2 had a failed intravenous access attempt, Group 3 had intravenous access established but did not use epinephrine, and Group 4 had intravenous access established and epinephrine administered. ^*^Four-group comparison analysis was conducted using a one-way analysis of variance (p < 0.05) and post-hoc analysis with the Scheffe test; Group 1 > Group 4 for age, Group 1 > Group 4 for RTI, Group 4 > Group 1 > Group 2 and 3 for STI, and Group 3 > Group 4 for TTI. CPR, cardiopulmonary resuscitation; AED, automated external defibrillator; RIT, response time interval; STI, scene time interval; TTI, transport time interval; TTM, targeted temperature management; SD, standard deviation.

Supplementary Table 2. Survival to discharge and favorable neurological outcomes of adult nontraumatic out-of-hospital cardiac arrest patients according to IV accessibility and prehospital epinephrine administration, 2020–2022

| **Survival to discharge** | | | | | |
| --- | --- | --- | --- | --- | --- |
|  | Survival | Unadjusted OR (95% CI) | p*-*value | Adjusted OR (95% CI) | p*-*value |
| Total (N = 1561) | 209 (13.4%) |  |  |  |  |
| Group 1 (N = 691) | 101 (14.6%) | Ref. | 0.008^*^ | Ref. | 0.068^*^ |
| Group 2 (N = 191) | 16 (8.4%) | 0.534 (0.307–0.929) | 0.026 | 0.613 (0.338–1.111) | 0.107 |
| Group 3 (N = 401) | 66 (16.5%) | 1.151 (0.821–1.614) | 0.415 | 1.061 (0.717–1.570) | 0.767 |
| Group 4 (N = 278) | 26 (9.4%) | 0.603 (0.382–0.950) | 0.029 | 0.594 (0.355–0.995) | 0.048 |
| **Favorable neurological outcomes** | | | | | |
|  | Favorable | Unadjusted OR (95% CI) | p*-*value | Adjusted OR (95% CI) | p*-*value |
| Total (N = 1560) | 82 (5.3%) |  |  |  |  |
| Group 1 (N = 690) | 36 (5.2) | Ref. | 0.003^*^ | Ref. | 0.013^*^ |
| Group 2 (N = 191) | 6 (3.1) | 0.589 (0.244–1.420) | 0.238 | 0.767 (0.277–2.128) | 0.611 |
| Group 3 (N = 401) | 34 (8.5) | 1.683 (1.035–2.736) | 0.036 | 1.156 (0.611–2.187) | 0.657 |
| Group 4 (N = 278) | 6 (2.2) | 0.401 (0.167–0.962) | 0.041 | 0.212 (0.075–0.601) | 0.004 |

The variables are presented as numbers of patients (percentages). The groups were divided based on intravenous accessibility and prehospital epinephrine use. Group 1 did not have intravenous access attempted, Group 2 had a failed intravenous access attempt, Group 3 had intravenous access established but did not use epinephrine, and Group 4 had intravenous access established and epinephrine administered. Neurological outcomes were scaled using cerebral performance categories, and categories 1 and 2 were defined as favorable neurological outcomes. ^*^Four-group comparison analysis was conducted using a chi-squared test. Other p-values represent significance level of the 95% confidence interval. The models were adjusted for potential confounding factors, including age, sex, comorbidities, witnessed status, arrest location, bystander CPR, bystander AED use status, initial rhythm, advanced airway management, mechanical chest compression use, and EMS processing time. OR, odds ratio; CI, confidence interval; CPR, cardiopulmonary resuscitation; AED, automated external defibrillator.

Supplementary Table 3. Survival to discharge and favorable neurological outcomes according to the initial rhythm at the scene of adult nontraumatic out-of-hospital cardiac arrest patients according to IV accessibility and prehospital epinephrine administration, 2020–2022

| **Shockable rhythm at the scene** | | | | | |
| --- | --- | --- | --- | --- | --- |
| **Survival to discharge** | | | | | |
|  | Survival | Unadjusted OR (95% CI) | p*-*value | Adjusted OR (95% CI) | p*-*value |
| Total (N = 220) | 87 (39.5%) |  |  |  |  |
| Group 1 (N = 83) | 34 (41.0%) | Ref. | 0.002^*^ | Ref. | 0.001^*^ |
| Group 2 (N = 21) | 3 (14.3%) | 0.240 (0.066–0.880) | 0.031 | 0.301 (0.074–1.235) | 0.096 |
| Group 3 (N =77) | 41 (53.2%) | 1.641 (0.878–3.069) | 0.121 | 1.904 (0.883–4.106) | 0.100 |
| Group 4 (N = 39) | 9 (23.1%) | 0.432 (0.182–1.026) | 0.057 | 0.288 (0.101–0.817) | 0.019 |
| **Favorable neurological outcomes** | | | | | |
|  | Favorable | Unadjusted OR (95% CI) | p*-*value | Adjusted OR (95% CI) | p*-*value |
| Total (N = 220) | 64 (29.1%) |  |  |  |  |
| Group 1 (N = 83) | 26 (31.3%) | Ref. | 0.006^*^ | Ref. | 0.001^*^ |
| Group 2 (N = 21) | 2 (9.5%) | 0.231 (0.050–1.065) | 0.060 | 0.245 (0.046–1.315) | 0.101 |
| Group 3 (N = 77) | 31 (40.3%) | 1.477 (0.771–2.830) | 0.239 | 1.173 (0.529–2.601) | 0.695 |
| Group 4 (N = 39) | 5 (12.8%) | 0.322 (0.113–0.919) | 0.034 | 0.116 (0.032–0.425) | 0.001 |
| **Nonshockable rhythm at the scene** | | | | | |
| **Survival to discharge** | | | | | |
|  | Survival | Unadjusted OR (95% CI) | p*-*value | Adjusted OR (95% CI) | p*-*value |
| Total (N = 1341) | 122 (9.1%) |  |  |  |  |
| Group 1 (N = 608) | 67 (11.0%) | Ref. | 0.174^*^ | Ref. | 0.645^*^ |
| Group 2 (N = 170) | 13 (7.6%) | 0.669 (0.360–1.243) | 0.203 | 0.789 (0.413–1.507) | 0.473 |
| Group 3 (N = 324) | 25 (7.7%) | 0.675 (0.418–1.092) | 0.109 | 0.753 (0.452–1.255) | 0.276 |
| Group 4 (N = 239) | 17 (7.1%) | 0.618 (0.355–1.077) | 0.089 | 0.768 (0.422–1.399) | 0.389 |
| **Favorable neurological outcomes** | | | | | |
|  | Favorable | Unadjusted OR (95% CI) | p*-*value | Adjusted OR (95% CI) | p*-*value |
| Total (N = 1340) | 18 (1.3%) |  |  |  |  |
| Group 1 (N = 607) | 10 (1.6%) | Ref. | 0.352^*^ | Ref. | 0.306^*^ |
| Group 2 (N = 170) | 4 (2.4%) | 1.439(0.445–4.645) | 0.543 | 2.502 (0.664–9.436) | 0.176 |
| Group 3 (N = 324) | 3 (0.9%) | 0.558(0.152–2.042) | 0.378 | 0.796 (0.171–3.700) | 0.771 |
| Group 4 (N = 239) | 1 (0.4%) | 0.251(0.032–1.970) | 0.188 | 0.346 (0.037–3.267) | 0.354 |

The variables are presented as numbers of patients (percentages). The groups were divided based on intravenous accessibility and prehospital epinephrine use. Group 1 did not have intravenous access attempted, Group 2 had a failed intravenous access but attempt, Group 3 had intravenous access established but did not use epinephrine, and Group 4 had intravenous access established and epinephrine administered. Neurological outcomes were scaled using cerebral performance categories, and categories 1 and 2 were defined as favorable neurological outcomes. ^*^Four-group comparison analysis was conducted using a chi-squared test. The models were adjusted for potential confounding factors, including age, sex, comorbidities, witnessed status, arrest location, bystander CPR, bystander AED use status, initial rhythm, advanced airway management, mechanical chest compression use, and EMS processing time. OR, odds ratio; CI, confidence interval; CPR, cardiopulmonary resuscitation; AED, automated external defibrillator.
